# COVID-19 – neue Herausforderungen in der Dysphagie- und Atemtherapie

**DOI:** 10.1007/s00115-021-01162-5

**Published:** 2021-07-09

**Authors:** Ulrike Frank, Katrin Frank

**Affiliations:** 1 Department Linguistik, Swallowing Research Lab, Universität Potsdam, Karl-Liebknecht-Str. 24–25, 14.202, 14476 Potsdam, Deutschland; 2 St. Vincenz Krankenhaus, Paderborn, Deutschland

**Keywords:** Long-COVID, Fatigue, Post Intensive Care Syndrome (PICS), Laryngeale Funktionen, Hypoxämie, Long COVID, Fatigue, Post intensive care syndrome (PICS), Laryngeal functions, Hypoxemia

## Abstract

Eine COVID-19-Erkrankung kann zu schweren Krankheitsverläufen mit multiplen Organbeteiligungen und respiratorischen und neurologischen Funktionseinschränkungen führen. Schluckstörungen (Dysphagien) können in dieser Patientengruppe durch primäre Schädigungen des zentralen und peripheren neuronalen Netzwerkes der Schluckfunktion entstehen, aber auch bedingt durch die häufig längere intensivmedizinische Behandlung und Beatmung. Erste klinische Befunde zeigen persistierende Dysphagien im Rahmen des Post-COVID-Syndroms („Long-COVID“), sodass die Patienten auch längerfristige Maßnahmen zur Rehabilitation einer sicheren und suffizienten oralen Nahrungsaufnahme benötigen. Daher sollte in die Behandlung von COVID-19-Patienten ein strukturiertes erkrankungsspezifisches Monitoring in Bezug auf Dysphagiesymptome integriert werden, und atemtherapeutische Maßnahmen zur Regulation von Husteneffektivität und Atem-Schluck-Koordination sollten auch bei diesen Patienten essenzieller Bestandteil des Dysphagiemanagements sein. Herausforderungen ergeben sich dabei einerseits durch die erforderlichen Anpassungen etablierter Behandlungsstandards an den Infektionsschutz. Zudem müssen Auswahl und Durchführungsintensität therapeutischer Maßnahmen an die Kapazitäten und die spezifische Pathophysiologie der COVID-19- und Long-COVID-Patienten angepasst werden, um weitere funktionelle Verschlechterungen zu vermindern.

## Abstract (en)

Coronavirus disease 2019 (COVID-19) can lead to severe disease courses with multiple organ involvement, respiratory and neurological functional impairments. Swallowing disorders (dysphagia) in this patient group can result from primary damage to the central and peripheral neuronal swallowing network but also from the frequently prolonged intensive care treatment and mechanical ventilation. Clinical observations indicate persistence of dysphagia in post-acute COVID-19 syndrome (long COVID), so that these patients probably also need long-term interventions for rehabilitation of safe and sufficient oral feeding. Therefore, structured disease-specific monitoring of dysphagia symptoms should be integrated into the treatment of COVID-19 patients and respiratory therapy should be an essential part of dysphagia management to re-establish cough effectiveness and breathing-swallowing coordination. Challenges arise from necessary adjustments to established treatment standards to prevent infections. Furthermore, the selection and intensity of therapeutic measures have to be adapted to the capacities and the specific pathophysiology of COVID-19 and long COVID patients to prevent further functional deterioration.

Die Behandlung von COVID-19-Patienten stellt das multidisziplinäre Dysphagieteam vor neue Herausforderungen. Vor allem bei schweren Krankheitsverläufen ist mit Beeinträchtigungen der Schluckfunktion (Dysphagien) zu rechnen, die durch die Erkrankung selbst und durch notwendige intensivmedizinische Behandlungen entstehen. Auch in Zukunft werden zunehmend Dysphagiepatienten behandelt werden, die unter Long-COVID-Symptomen leiden.

In diesem Beitrag werden im ersten Teil Symptomatik, Verlauf und Behandlung bei COVID-19-Patienten in Bezug auf die Relevanz für die Entstehung von Schluckstörungen (Dysphagien) beschrieben. Die Angaben und Handlungsempfehlungen (Stand März 2021) stammen aus Publikationen des Robert-Koch-Instituts (RKI), der Deutschen Interdisziplinären Vereinigung für Intensiv- und Notfallmedizin (DIVI), der Deutschen Gesellschaft für Neurologie (DGN), der Deutschen Gesellschaft für Pneumologie und Beatmungsmedizin (DGP) sowie der einschlägigen Dysphagiefachgesellschaften: Dysphagia Research Society (DRS), European Society for Swallowing Disorders (ESSD), Deutsche interdisziplinäre Gesellschaft für Dysphagie (DGD), Royal College of Speech Language Therapists – RCSLT (UK), National Tracheostomy Safety Project – NTSP (UK).

Im zweiten Teil werden spezifische atemtherapeutische Maßnahmen bei COVID-19 und Long-COVID erläutert, diese beruhen auf ersten klinischen Erfahrungen.

## Symptomatik und Verlauf

COVID-19-Infektionen gehen mit einer großen Bandbreite möglicher Symptome einher, die einen direkten oder indirekten Einfluss auf die Schluckfunktion und die orale Ernährung haben können. Die Symptome zeigen sich im Mittel erstmals nach 5 Tagen. Einige, wie z. B. Husten, Fieber und Schnupfen weisen offensichtlich auf eine Infektion des respiratorischen Traktes hin, andere sind unspezifischer, wie z. B. Geschmacks- und Geruchsbeeinträchtigungen (Ageusie, Anosmie), Übelkeit und Erbrechen und Herzrhythmusstörungen, Blutgerinnungsstörungen sowie kardiale Dekompensationen [22]. Neuere Studien zeigen, dass es bei COVID-19-Infektionen auch zu laryngealen Ödemen, Laryngitis und Paresen kommen kann [8, 19, 28], sodass der endoskopischen Schluckuntersuchung bei der Dysphagiediagnostik und im Trachealkanülenmanagement ein besonderer Stellenwert zukommt.

Dass SARS-CoV‑2 kein reines Atemwegsvirus ist, zeigt sich auch in der Konzentration der Viruslast pro Zelle in unterschiedlichen Organen. Diese ist zwar in den Atemwegen am höchsten, jedoch finden sich Nachweise von SARS-CoV‑2 auch in Nieren, Herz, Leber, Gehirn und Blut. Auftretende Enzephalopathien stehen im Zusammenhang mit erhöhter Letalität und Morbidität, jedoch besteht kein klarer Zusammenhang zwischen SARS-CoV-2-Befunden in unterschiedlichen Hirnregionen und den Symptomen einer Neuroinflammation, sodass sowohl eine Autoimmunenzephalitis als auch die SARS-CoV-2-Infektion direkt (para- bzw. postinfektiös) als Ursache infrage kommen [17]. Da neurologische Manifestationen der SARS-CoV-2-Infektion leicht durch die kritische respiratorische Situation maskiert werden können, sollte eine spezifische Diagnostik zur Aufdeckung von Schädigungen des zentralen und peripheren Nervensystems erfolgen [2, 14].

Die Verlaufsformen der Erkrankung können sehr unterschiedlich sein: von asymptomatischen und leichteren Verläufen bis hin zu schweren Verläufen mit Hypoxämien, Dys- und Tachypnoe (>30 AZ/min), Lungeninfiltraten und Pneumonien. Schwere und kritische Krankheitsverläufe sind häufig durch eine plötzliche Verschlechterung innerhalb weniger Stunden gekennzeichnet und führen zu schweren Pneumonien bis hin zu akutem Lungenversagen („acute respiratory distress syndrome“, ARDS) und Multiorganversagen (Sepsis; [1]).

Der Anteil schwerer und kritischer Verläufe liegt bei ca. 15–20 %, ca. 5 % dieser Patienten werden intensivmedizinisch behandelt und häufig längerfristig (>72 h) intubiert beatmet [12]. Die Mortalitätsrate liegt mit 1,6 % mehr als 10-fach höher als bei einer Grippeinfektion (0,1 %), dabei steigt die Rate bei hospitalisierten COVID-19-Patienten auf 22 %, bei beatmeten Patienten ist sie mehr als 3‑fach erhöht gegenüber nichtbeatmeten Patienten [12].

Zum Langzeitverlauf („Long-COVID“) gibt es derzeit noch wenige gesicherte Erkenntnisse. Es zeigt sich aber, dass Symptome auch nach leichten Verläufen noch Wochen bis Monate nach der akuten Erkrankung (neu) auftreten können. Nach ersten Einschätzungen werden bis zu 50 % der hospitalisierten Patienten längerfristig Unterstützung benötigen [18]. Zunehmend wird auch das Auftreten eines Guillian-Barré-Syndroms (GBS) in der Akutphase und als Langzeitkomplikation (Post-COVID-GBS) beschrieben [2, 27]. Bereits bekannte langfristige Folgen umfassen Erschöpfungs- und Fatiguezustände mit Dyspnoe und Tachypnoe („postintensive care syndrome“, PICS), die die Schluckfunktion beeinträchtigen können [4], sowie Kopfschmerzen, Schwindel, Konzentrations- und Gedächtnisstörungen. Hinzu kommen neuropsychiatrische Beeinträchtigungen wie Depressionen, Angststörungen und Schlafstörungen [16] mit erheblicher Minderbelastbarkeit und Einschränkungen der Lebensqualität, der Partizipationsfähigkeit und der beruflichen Perspektiven der häufig noch relativ jungen COVID-19-überlebenden Patienten.

## Intensivmedizinische Maßnahmen bei COVID-19-Patienten

Hauptziel in der Behandlung akut hypoxämischer COVID-19-Patienten ist die Sicherstellung einer ausreichenden Oxygenierung. Hierzu wird zunächst ein Therapieversuch mit Sauerstoff („high-flow nasal oxygen“, HFNO) unter kontinuierlichem Monitoring und Intubationsbereitschaft durchgeführt. Kann hierdurch keine ausreichende Sättigung erreicht werden (SpO_2_ ≤90 %, bei COPD <88 %, PaO_2_ <55 mmHG, ph ≥7,35), so ist eine nichtinvasive Beatmung (NIV) indiziert [10]. Probleme können sich bei NIV-Beatmung durch die erhöhte Gefahr der Aeorosolverbreitung ergeben, aber auch dadurch, dass die häufig wachen und vigilanten Patienten einen erheblichen Leidensdruck erleben, insbesondere durch „Lufthunger“ und das bewusste Erleben von Isolation und Einsamkeit. Hinzu kommt das Bewusstsein bez. der schlechten Prognose für intensivpflichtige COVID-19-Patienten. Die spezielle ITS-Umgebung, die nur wenig Identifikationspunkte bietet, trägt zur Orientierungslosigkeit und zum Entstehen von Delirzuständen der Patienten bei. Diese erschweren die Kommunikationsmöglichkeiten und die Durchführung einer strukturierten Befundung und Wiederherstellung der oralen Ernährung.

Entwickelt sich im Verlauf ein ARDS und Multiorganversagen, ist eine invasive Beatmung mit hohem Beatmungsdruck, hohem PEEP und hochdosierter O_2_-Gabe über mindestens 16 h erforderlich (sog. „aggressive Beatmung“; [10, 13]), was von den schwersterkrankten und kardial insuffizienten Patienten häufig nur schlecht toleriert wird und in Bezug auf eine intrakranielle Druckerhöhung problematisch sein kann [2, 5, 6, 25]. Auch eine effektive Mundpflege ist in Bauchlage erschwert, sodass bei einer erhöhten intraoralen Keimbelastung und Speichelaspiration das Pneumonierisiko erhöht sein kann.

Grundsätzlich sollte auch bei COVID-19-Patienten ein früher Wechsel vom Tubus auf invasive Beatmung über eine Trachealkanüle erfolgen, da hierdurch die Weaningzeit verkürzt, Sedativa vermindert und Komplikationen verringert werden können [11]. Allerdings sind Extubationsversuche in dieser Patientengruppe mit einem hohen Risiko von Reintubationen und Aerosolbildung verbunden, sodass Einzelfallentscheidungen getroffen und die Extubation ggf. erst nach dem 21. Beatmungstag durchgeführt wird [2, 3]. Die Beatmungsdauer hängt vom Schweregrad des Krankheitsverlaufs ab, im Mittel beträgt sie 13,5 Tage [12], damit wird die sonst durchschnittliche Dauer invasiver Beatmung von 7 bis 10 Tagen häufig überschritten.

Zusammenfassend sind bei COVID-19-Patienten sowohl die Gesamtdauer der intensivmedizinischen Behandlung als auch die Intubations- und Beatmungsdauer erhöht [7, 24]. Hierdurch steigt die Gefahr dysphagierelevanter Folgekomplikationen.

## Dysphagiemanagement bei COVID-19-Patienten

Eine aspirationsfreie und suffiziente orale Nahrungsaufnahme setzt intakte Funktionen des komplexen zentralen und peripheren neurophysiologischen Netzwerks der Schluckfunktion voraus. Auf jeder dieser Ebenen kann durch eine COVID-19-Erkrankung ein Funktionsverlust entstehen, der zu einer Dysphagie führt [9]:

- spezifische kortikale und Hirnstammareale und kortikobulbäre Bahnen: Schädigung durch Enzephalopathien, Schlaganfall;
- Hirnnerven (V, VII, IX, X und XII) und Spinalnerven (C1–C3): CIP, CIM, Guillain-Barré-Syndrom;
- schluckspezifische oropharyngeale Muskulatur und sensible Rezeptoren: Verletzungen (z. B. durch Intubation), entzündliche Prozesse.

Post-Extubations Dysphagien (PED), verbunden mit einer hohen Rate an (stillen) Aspirationen, sind insbesondere aufgrund der oben beschriebenen verlängerten Intubations- und Beatmungszeiten zu erwarten [8, 15, 19, 30]. Die mit der Langzeitintubation verbundene Sedierungsmedikation kann sich zusätzlich negativ auf die Schluckfunktion und kognitive Fähigkeiten auswirken [29]. Eine erste Fallserienstudie zeigte, dass auch der Zeitpunkt der Dekanülierung bei kritisch kranken COVID-19-Patienten verzögert sein kann [9].

Eine zentrale Problemstellung im Dysphagiemanagement bei akut erkrankten COVID-19-Patienten stellt das hohe Infektionsrisiko durch Aerosolbildung dar, die bei vielen Maßnahmen gegeben ist z. B. durch forcierte Ausatmung, Nies- oder Hustenreaktionen [20]. Das Infektionsrisiko ist bei dysphagietherapeutischen Interventionen auch dadurch erhöht, dass der Mindestabstand nicht eingehalten werden kann und der Patient bei vielen Interventionen keinen Mund-Nasen-Schutz trägt. Die Maßnahmen im Dysphagiemanagement müssen daher an empfohlene Hygieneschutzmaßnahmen angepasst werden (Tab. 1; [20, 29]).

**Table 1:** 

Persönliche Schutzausrüstung (PSA)	„Minimal team approach“	Anpassung der Interventionen
– Schutzhandschuhe – Schutzkittel – Atemschutzmaske (mind. FFP2) – Augenschutz – Haarschutz – Schuhschutz	– Nur zwingend notwendige Personen im Raum – Geeignete Maßnahmen werden professionsunabhängig durchgeführt – Kritische Maßnahmen werden von der Person mit der größten Expertise durchgeführt	– Risiko-Nutzen-Abwägung: AGPs nur bei eindeutigem Nutzen und mit PSA *Wenn möglich:* – Geschlossenes Absaugsystem – Mindestabstand – Telemedizinische Maßnahmen

Insbesondere beim Trachealkanülenmanagement und bei endoskopischen Schluckuntersuchungen sind in der Akutversorgung Abweichungen von etablierten Behandlungsstandards erforderlich [20, 28]. Dennoch sprechen sich die aktuellen Empfehlungen der einschlägigen Fachgesellschaften zum risikoadaptierten Vorgehen bei COVID-19-Patienten dafür aus, auf eine strukturierte bildgebende Schluckuntersuchung nicht zu verzichten und therapeutische Maßnahmen zum TK-Weaning mit dem Ziel einer Dekanülierung mit folgenden Anpassungen durchzuführen [20, 21, 29]:

- Begrenzung von Frequenz und Dauer der Entblockungsversuche;
- Entblocken bei beatmeten Patienten vorzugsweise in Spontanatemphasen, um Aerosolverbreitung durch den Respiratordruck zu vermeiden;
- Abdecken der entblockten TK mit einem Mund-Nasen-Schutz, wenn der Patient dies toleriert und eine ausreichende Atmung sichergestellt wird;
- Verwendung von Trachealkanülen mit subglottischer Absaugmöglichkeit, Absaugen über geschlossenes System;
- Maßnahmen zur „above cuff vocalization“ (ACV) sollten minimiert werden; die Durchführbarkeit ist angesichts der laryngealen Entzündungen und Ödeme ohnehin fraglich.

Weitere Problemstellungen ergeben sich in der Dysphagiebehandlung durch die teils persistierenden (>6 Monate) respiratorischen, neuromuskulären und neurologisch-kognitiven Beeinträchtigungen, die das klinische Bild der Dysphagie verschlechtern und den Rehabilitationserfolg reduzieren ([4, 26]; Tab. 2). Es ist mit einer hohen Prävalenz von laryngealen Entzündungen, Ödemen und Paresen zu rechnen. Erste Daten zu Dysphonien weisen eine Prävalenz von ca. >60 % in der Akutsituation und 37 % bei Long-COVID-Patienten auf, die Dysphagierate wird mit >27 % bei Long-COVID beziffert [23]. Der flexiblen endoskopischen Schluckuntersuchung (FEES) kommt hierbei eine besondere Bedeutung zu, vor allem da die laryngealen Komplikationen ohne eine bildgebende Untersuchung häufig unentdeckt bleiben [8, 28].

**Table 2:** 

Pathomechanismus	Folge
– Längere Beatmungs- und Intubationszeiten – Erschwerte Intubation/Reintubationen – Delir	– Post-Intubations Dysphagie (PED) – Hohe Prävalenz von (stillen) Aspirationen – Ggf. manifeste laryngeale Schädigungen – Atem-Schluck-Dyskoordination – Verminderte Therapiecompliance
Dysfunktionale Atemmuskulatur (z. B. aufgrund CIP/CIM)	– Ineffektives Husten bei Penetration/Aspiration – Erhöhtes Pneumonierisiko (VAP) – Atem-Schluck-Dyskoordination
Laryngitis/Larynxödeme	– Dysfunktionale laryngeale Funktionen: Atemwegschutz, Phonation, Husten – Erschwerte endoskopische Befundung der Dysphagie – Schmerzen – Erschwertes TK-Weaning
Geruchs‑/Geschmackseinschränkungen	– Inappetenz, verminderte Nahrungs- und Nährstoffaufnahme – Verminderte Effektivität olfaktorischer und gustatorischer Stimulationen in der Dysphagietherapie – Erschwerter oraler Kostaufbau

*PED* Post-Extubations Dysphagie, *CIP* Critical-illness-Polyneuropathie, *CIM* Critical-illness-Myopathie, *VAP* „ventilator-associated pneumonia“

Bei allen dysphagietherapeutischen Maßnahmen sollten neue Risikofaktoren und Problemstellungen beachtet werden, die mit der COVID-19-Erkrankung einhergehen:

- Bei der Mobilisierung, Lagerung und Positionierung ist mit O_2_-Abfällen und kardialer Instabilität zu rechnen.
- Überlastungen des Patienten tragen zur Fatigue bei und reduzieren die Effektivität weiterer Maßnahmen.
- Insbesondere beim Absaugen und bei endoskopischen Interventionen besteht erhöhte Gefahr durch Blutgerinnungsstörungen.
- Delirzustände können das Situationsverständnis und die Compliance erheblich einschränken und spezifische Maßnahmen erschweren.
- Beeinträchtigungen der Geschmacks- und Geruchswahrnehmung sowie Mundtrockenheit werden die Möglichkeiten zur Schluckstimulationen mit Nahrung und den oralen Kostaufbau beeinträchtigen.

Das dysphagietherapeutische Ziel, einen möglichst weitgehenden und sichern oralen Kostaufbau zu erreichen, wird bei den kritisch kranken COVID-19-Patienten unter anderem durch die hohen Aspirationsraten, ineffektives protektives Husten, die dysfunktionale Atmung und die Atem-Schluck-Dyskoordination erschwert. Daher sollten auch bei COVID-19-Patienten atemtherapeutische Maßnahmen fester Bestandteil des Dysphagiebehandlung sein.

## Atemtherapie bei COVID-19-Patienten

Atemtherapie ist bei COVID-19-Patienten in allen Erkrankungsphasen indiziert. Im Vordergrund stehen dabei Maßnahmen zur Mobilisierung der Atemmuskulatur und zur Normalisierung von Atemfrequenz und Atemvolumen und mit dem Ziel, die Aspirationsgefahr zu vermindern, die Husteneffektivität zu verbessern und die Atem-Schluck-Koordination zu regulieren. Grundsätzlich sind zahlreiche etablierte atemtherapeutische Lagerungs‑, Hands-on- und aktive Atemtechniken geeignet, es müssen jedoch Anpassungen im Vorgehen erfolgen, um der speziellen Pathophysiologie der Patienten gerecht zu werden.

Eines der Hauptprobleme bei COVID-19 ist die Hypoxämie, die teils auch als „stille Hypoxämie“ auftreten kann, sodass die Patienten subjektiv keine Luftnot empfinden und berichten. Eine Überlastung und Erschöpfung beispielsweise durch eine intensive Übungstherapie, Frühmobilisation oder durch Entblockungsintervalle werden daher vom Patienten ggf. nicht zurückgemeldet und es besteht die Gefahr von Organschädigungen und Funktionsverlusten.

Daher sollten bevorzugt atemtherapeutische Methoden einbezogen werden, die wenig Stress und Belastung erzeugen. Auf aktive Atemübungen sollte nicht verzichtet werden, da sie effektiver als passive Methoden sind und der Therapeut besser einen Sicherheitsabstand wahren kann.

Beispiele für geeignete atemtherapeutische Maßnahmen bei akut erkrankten und Long-COVID-Patienten illustriert Tab. 3. Die Auswahl der Methoden sollte indikationsspezifisch erfolgen, bei akut erkrankten, infektiösen Patienten müssen Anpassungen entsprechend der oben erläuterten Infektionsschutzmaßnahmen erfolgen (Tab. 1).

**Table 3:** 

Lagerung – Positionierung	Hands-on-Techniken	Aktive Atemtechniken
*Ziele:*		
– Verbesserung des Ventilations-Perfusions-Verhältnisses (Prinzip „good lung down“) – Thoraxmobilisation und Tonusregulation der Atemmuskulatur	– Gewebelockerung und Tonusregulation – Aktive Atemwahrnehmung und -steuerung – Rezeptoraktivierung	– Aktive Steigerung des Atemvolumens – Erhöhung der Übungsintensität und Eigenaktivität – Ggf. Einüben von Kompensationsstrategien
*Beispiele:*		
– Bauchlage oder Sitz mit Oberkörpervorlage – >90°-Seitenlage – Dreh-Dehn-Lagerungen/C-Lagerung – Thoraxweitstellung mit/ohne Rotation	– Kontakt- und Richtungsatmen – Mobilisation des Thorax und der Rippengelenke im Atemrhythmus – Reiz- und Packegriffe – Physikalische und thermische Anwendungen: Vibrax, „heiße Rolle“ – Reflektorische Atemtherapie	– Aktives „air stacking“ – Umgekehrte Intervallatmung – Pfeil-und-Bogen-Technik – Sniffing – Summendes Ausatmen

## Hustenmanagement

COVID-19-Patienten sollen einen Hustenreiz nicht generell unterdrücken, auch wenn eine hohe Gefahr der Aerosolverbreitung und Verstärkung der Fatigue gegeben ist. Ziel der atemtherapeutischen Interventionen ist, mit den Patienten Strategien für ein effektives, produktives Husten zu erarbeiten, sodass längere unproduktive Hustensalven mit einer entsprechend hohen Aerosolstreuung und Erschöpfung des Patienten vermieden werden. Strategien für effektives Husten sind z. B.

- Husten im Kutschersitz mit einwärtsgedrehten Armen,
- bewusste tiefe Einatmung und aktive Verstärkung der Kompressionsphase (Bauchpresse),
- Techniken zur Hustenreizhemmung, wie z. B. Umgekehrte Intervallatmung oder das Ausatmen in die geschlossene Faust oder mit Lippenbremse.

Die beschriebenen atemtherapeutischen Maßnahmen sowie rumpfaktivierende und -mobilisierende Übungen tragen mittelfristig zur Erhöhung des Inspirationsvolumens und zur Verbesserung der Kompressionsphase und damit auch zur Verbesserung der Husteneffektivität bei.

## Fazit für die Praxis

- In die Behandlung von COVID-19-Patienten sollte ein strukturiertes Monitoring in Bezug auf Dysphagiesymptome integriert werden.
- Atemtherapeutische Maßnahmen sind im Dysphagiemanagement bei COVID-19- und Long-COVID-Patienten essenziell.
- Ziel der Atemtherapie ist, das Atemvolumen zu erhöhen, die Husteneffektivität zu verbessern und die Atem-Schluck-Koordination zu regulieren.
- Die Intensität therapeutischer Maßnahmen muss an die Kapazitäten des Patienten angepasst (ggf. reduziert) werden, um Überlastung zu vermeiden.

## References

[1] Bein B, Bachmann M, Hugett S (2020). SARS-CoV-2/COVID-19: Empfehlungen zu Diagnostik und Therapie. Anasthesiol Intensivmed Notfallmed Schmerzther.

[2] Berlit P et al (2021) Neurologische Manifestationen bei COVID-19, S1-Leitlinie. www.dgn.org/leitlinien. Zugegriffen: 6. März 2021 (Deutsche Gesellschaft für Neurologie (Hrsg.), Leitlinien für Diagnostik und Therapie in der Neurologie)

[3] Brodsky MB, Nollet J, Spronk PE (2020). Prevalence, pathophysiology, diagnostic modalities and treatment options for dysphagia in critically ill patients. Am J Phys Med Rehabil.

[4] Brodsky M, Huang M, Shanholtz C (2016). Recovery from dysphagia symptoms after oral endotracheal intubation in acute respiratory distress syndrome survivors. A 5-year longitudinal study. Ann Am Thorac Soc.

[5] Cook TM, El-Boghdadly K, McGuire B (2020). Consensus guidelines for managing the airway in patients with COVID-19: guidelines from the Difficult Airway Society, the Association of Anaesthesists, the Intensive Care Society, the Faculty of Intensive Care Medicine and the Royal College of Anaesthesists. Anaesthesia.

[6] Coppo A, Bellani G, Winterton D (2020). Feasibility and physiological effects of prone positioning in non-intubated patients with acute respiratory failure due to COVID-19 (PRON-COVID): a prospective study. Lancet Respir Med.

[7] Deutsche interdisziplinäre Vereinigung für Intensiv- und Notfallmedizin (2021) DIVI Intensivregister. www.intensivregister.de. Zugegriffen: 9. März 2021

[8] Dziewas R, Hufelschulte LM, Lepper J (2021). Dysphagia in patients with severe Coronavirus disease 2019—potential neurologic etiologies. Crit Care Explor.

[9] Dziewas R, Warnecke T, Zürcher P (2020). Dysphagia in COVID-19—multilevel damage to the swallowing network?. Eur J Neurol.

[10] Fichtner F, Mörer O, Laudi S et al (2017) S3-Leitlinie „Invasive Beatmung und Einsatz extrakorporaler Verfahren bei akuter respiratorischer Insuffizienz“. https://www.awmf.org/uploads/tx_szleitlinien/001-021l_S3_Invasive_Beatmung_2017-12.pdf. Zugegriffen: 9. März 2021 (DIVI, 4: 154–163)

[11] Griffiths RD, Hall JB (2010). Intensive care unit-acquired weakness. Crit Care Med.

[12] Karagiannidis C, Mostert C, Hentschker C (2020). Case characteristics, resource use, and outcomes of 10021 patients with COVID-19 admitted to 920 German hospitals: an observational study. Lancet Respir Med.

[13] Kluge S et al (2021) S3-Leitlinie – Empfehlungen zur stationären Therapie von Patienten mit COVID-19. https://www.awmf.org/leitlinien/detail/ll/113-001.html. Zugegriffen: 28. Febr. 2021 10.1055/a-1334-1925 33450783

[14] Macht M, White SD, Moss M (2014). Swallowing dysfunction after critical illness. Chest.

[15] Macht M, King CJ, Wimbish T, Clark BJ (2013). Post-extubation dysphagia is associated with longer hospitalization in survivors of critical illness with neurologic impairment. Crit Care.

[16] Mao L, Jin H, Wang M (2020). Neurologic manifestations of hospitalized patients with Coronavirus disease 2019 in Wuhan, China. JAMA Neurol.

[17] Matschke J, Lütgehetmann M, Hagel C (2020). Neuropathology of patients with COVID-19 in Germany: a post-mortem case series. Lancet Neurol.

[18] Maxwell E (2020) Living with COVID-19. A dynamic review of the evidence around ongoing COVID-19 symptoms (often called long COVID). https://evidence.nihr.ac.uk/themedreview/living-with-covid19/. Zugegriffen: 13. März 2021 (National Institute for Health Research (UK))

[19] McGrath B, Wallace S, Goswamy J (2020). Laryngeal oedema associated with COVID-19 complicating airway management. Anaesthesia.

[20] Miles A, Connor NP, Desai RV (2020). Dysphagia care across the continuum: a multidisciplinary dysphagia research society taskforce report of service-delivery during the COVID-19 global pandemic. Dysphagia.

[21] National Tracheostomy Safety Project (2020) COVID-19 response: safe tracheostomy care—a toolkit for healthcare staff, version 2. http://www.tracheostomy.org.uk/storage/files/Safe%20TrachyCareToolkit%20V9b.pdf. Zugegriffen: 13. März 2021

[22] Paterson RW, Brown RL, Benjamin L (2020). The emerging spectrum of COVID-19 neurology: clinical, radiological and laboratory findings. Brain.

[23] Regan J, Walshe M, Lavan S (2021). Post-extubation dysphagia and dysphonia amongst adults with COVID-19 in the Republic of Ireland: a prospective multi-site observational cohort study. Authorea.

[24] Robert Koch-Institut (2021) Epidemiologischer Steckbrief zu SARS-CoV‑2 und COVID-19. https://www.rki.de/DE/Content/InfAZ/N/Neuartiges_Coronavirus/Steckbrief.html. Zugegriffen: 1. März 2021

[25] Saccheri C, Morawiec E, Delemazure J (2020). ICU-acquired weakness, diaphragm dysfunction and long-term outcomes of critically ill patients. Ann Intensive Care.

[26] Schefold JC, Berger D, Zürcher P (2017). Dysphagia in mechanically ventilated ICU patients (DYnAMICS): a prospective observational trial. Crit Care Med.

[27] Toscano G, Palmerini F, Ravaglia S (2020). Guillain-Barré syndrome associated with SARS-CoV-2. N Engl J Med.

[28] Wallace S et al (2020) Speech and language therapy for COVID-19 patients in ICU and beyond. https://members.ics.ac.uk/ICS/ICS/Pdfs/COVID-19/Speech_and_language_therapy_for_COVID-19_patients_in_ICU_and_beyond.aspx. Zugegriffen: 9. März 2021 (Intensive Care Society)

[29] Zaga CJ, Pandian V, Brodsky MB (2020). Speech-language pathology guidance for tracheostomy during the COVID-19 pandemic: an international multidisciplinary perspective. Am J Speech Lang Pathol.

[30] Zuercher P, Moret CS, Dziewas R (2019). Dysphagia in the intensive care unit: epidemiology, mechanisms, and clinical management. Crit Care.

